# Intermacromolecular Interaction Determines the Long-Ranged Force and Self-Assembly of Microgels at the Air/Water Interface

**DOI:** 10.1021/acsmacrolett.5c00111

**Published:** 2025-04-22

**Authors:** Wei Liu, Zuwei Zhao, Li Zhang, Kangle Zhou, Pui Wo Felix Yeung, Hang Jiang, Cheng Yang, Yuwei Zhu, To Ngai

**Affiliations:** † The Key Laboratory of Synthetic and Biological Colloids, Ministry of Education and School of Chemical and Material Engineering, 66374 Jiangnan University, Wuxi 214122, China; ‡ Department of Chemistry, 26451 The Chinese University of Hong Kong, Shatin, N.T., Hong Kong 999077, China

## Abstract

We experimentally investigate the contribution of the interchain interaction to the interfacial stress and self-assembly of microgels at the air/water interface. Our results suggest that the intercorona penetrations contribute to an entropy-driven long-ranged force. The structural parameter and binding energy between neighboring microgels are given by using the radial distribution function, which further clarifies the intercorona and intercore interactions during the 2D phase transition.

## Graphical abstract

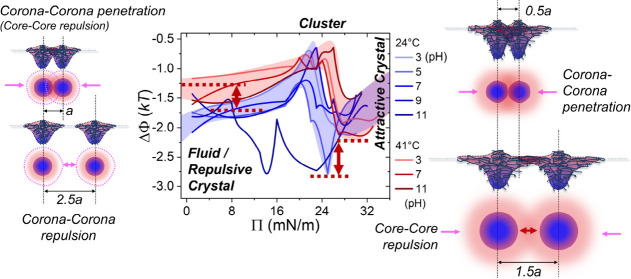

The delicate interplay of interchain interactions between soft polymeric entities at the fluid interface influences the dynamics, organization, and phase behaviors of systems from nanometric to macroscopic length scales, giving rise to a variety of ubiquitous phenomena such as interfacial self-assembly.^1–4^ Our current understanding and intuition of the interaction between microsized hydrogels (i.e., microgels) at the interface are grounded in central principles mainly from two parts: (a) chain entanglement, penetration, and percolation between the highly stretched corona at the oil or air side and (b) steric hindrance and classical DLVO forces, such as the electrostatic double-layer (EDL) repulsion between the charged and hydrophilic subjects in aqueous phase.^5–11^ These interactions involved at different length scales counteract each other, giving rise to stable long-range ordering at interfaces.

In-situ observations, including the interfacial core–corona topology, self-assembly, coexistence of ordered and disordered phase, nucleation of two-dimensional (2D) hexagonal crystal, nonuniform polycrystalline orientation, and solid–solid phase transition, have provided substantial validations of the microscopic structure and cooperative arrangement for arrested 2D materials composed of the soft thus deformable microgels.^12–19^ Moreover, indirect measurements such as interfacial tension in Pickering emulsion and surface pressure (Π) at the air/water interface have shed light on the dynamic interfacial behavior and instantaneous responsiveness of microgels to external stimuli such as the pH and temperature.^20–26^ Although this inspiring evidence has been achieved for years, clear and consistent reports of intermacromolecular interaction under interfacial confinement from both spatiotemporal and energic aspects have evaded explanation. Undoubtedly, extracting the key physics behind broadly distributed interfacial dynamics and mechanics remains a complicated task.

In this Letter, we focus on two primary questions: (a) first, from the viewpoint of length scale, how the ordered and disordered packing of soft microgels at the interface makes a response to external stimuli such as pH or temperature; and (b) from an energetic standpoint, how the self-assembly structure or clustering evolution correlates to the intermacromolecular interactions with a distinguishment of the contributions from the core or corona counterpart, particularly under varied stress, pH, and temperature simultaneously.

To do so, we study a simple but well-established model system, poly­(*N*-isopropylacrylamide-*co*-methacrylic acid) (p­(NIPAM-*co*-MAA)) microgels, which consist of a thermoresponsive cross-linked polymeric network incorporated with pH-sensitive carboxylic acid groups.^27^ The polymer PNIPAM is famous for its volume phase transition from a swollen to a collapsed state across a lower critical solution temperature of ∼32 °C in aqueous solution.^28–31^ Bringing in an ionic comonomer leads to additional swelling behavior because of the repelling interaction between the charged domains and the osmotic pressure of the counterions.^32–34^ Such pH–temperature dual-responsive microgels have been demonstrated for rapid adsorption at an oil/water or air/water interface, which can efficiently lower down the interfacial tension, thus stabilizing the interface, manifesting widespread prospects in many industrial areas.^2,4,14,21,33^

Here we report on the experimental findings of the contribution of intermacromolecular interaction to a long-ranged force and self-assembly of microgels at an air/water interface. We observe a universal long-ranged ordering (interaction) of microgels at low Π and an attractive crystallization at high Π. The corona–corona bridging effect combining a core–core repulsive interaction is proposed to attribute to the interfacial stress transfer, thus strengthening the ordered packing of microgels at the interface. Furthermore, we will show that the interfacial self-assembly and the nearest interparticle neighbor distance are predominately determined by the coordination of interfacial stress and temperature, rather than pH. The physics governing the mechanism is further elaborated by an energy landscape and phase diagram using a variety of interfacial- and solution-dependent parameters.

The p­(NIPAM-*co*-MAA) microgels of ca. 0.5 μm at room temperature were synthesized via an aqueous free radical precipitation polymerization.^7^ Increasing temperature can efficiently reduce the hydration ability (or hydrophilicity) of the PNIPAM-enriched macromolecular chains, driving microgels to expel water, thus, deswelling, particularly beyond the LCST. The reduced volume further caused an enhancement of surface charge density (≥40 °C; Figure 1a). On the other hand, increasing pH (>7) can make the MAA groups deprotonate, giving rise to a more negatively charged network. The additional charges induced a further swelling of microgels due to the interchain EDL repulsion and the osmotic pressure of the counterions (Figure 1b). Besides, increasing pH can subsequently enhance the wettability of microgels, showing consistency with the enhanced degree of ionization of the polymeric chains (Figures 1c and S1).

**Figure 1:**
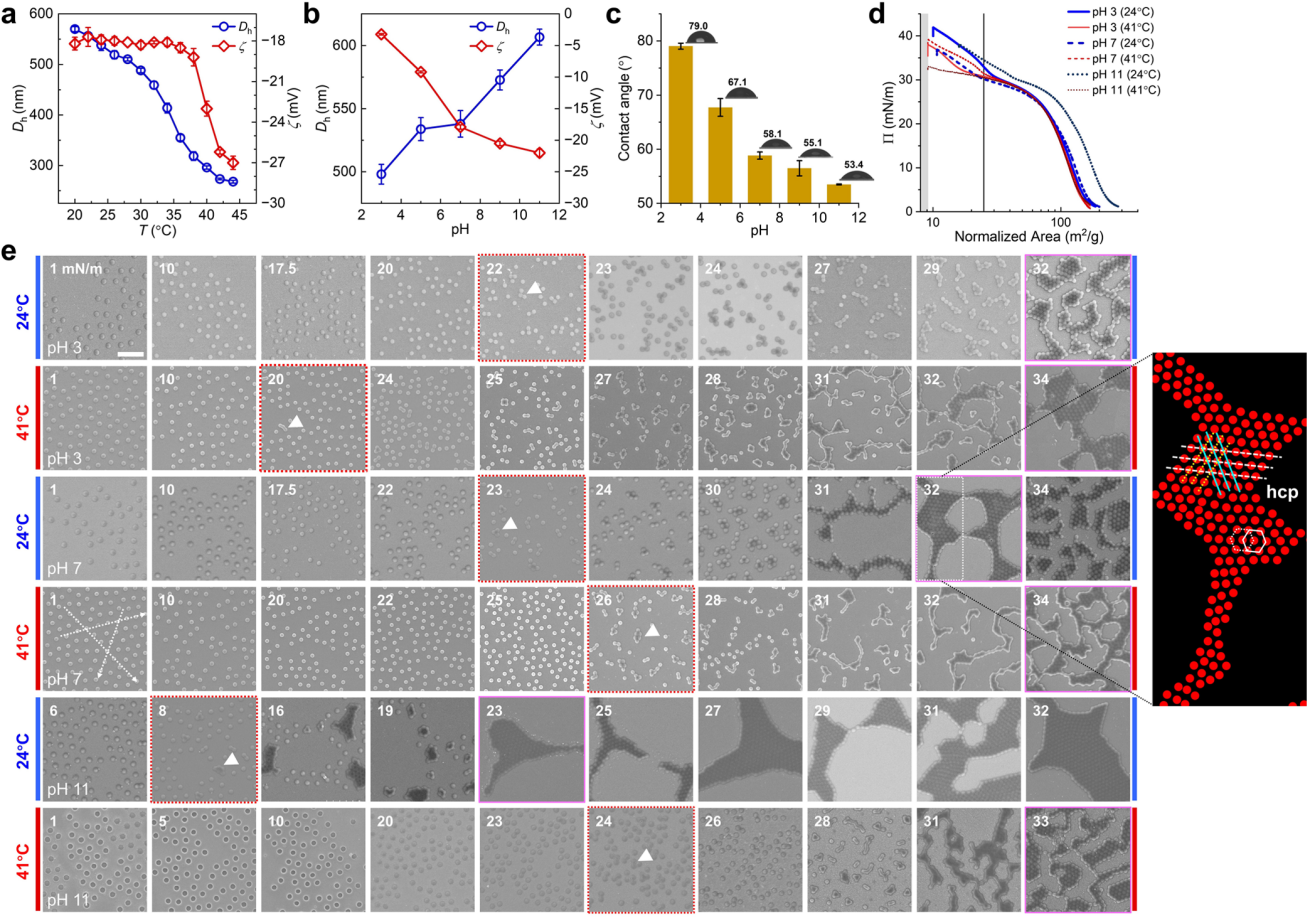
Swelling and interfacial behavior of the p­(NIPAM-*co*-MAA) microgels. Hydrodynamic diameter (*D*_h_) and zeta potential (ζ) vs temperature (a) and pH (b). (c) Water contact angles. (d) Compression isotherms. (e) SEM images of the self-assembly of microgels at the air–water interface after transfer to solid substrates. Scale bar: 2 μm. The magnitude of the surface pressure in mN/m is indicated at the upper-left in each panel. Red dotted and pink solid boxes denote clustering initiation (triangles) and attractive colloidal crystal formation, respectively. Right: regenerated array structure indicating a 2D hexagonal close-packed (hcp) lattice.

We first examined the compression isotherms of microgels spread at an air/water interface in a Langmuir trough, where the trough area was normalized by the amount of microgels at the interface (Figures 1d and S2). The normalized isotherms displayed a two-stage compression behavior for all conditions settled here, showing certain similarities with previous reports.^7,10^ Upon compression, the first steep increase of the surface pressure has been suggested as a result of the beginning of the corona–corona contact. We noticed that a pseudoplateau emerged at Π = 25–30 mN/m, which is almost accompanied by the concurrent formation of clusters, indicated by a direct observation where microgels at interface have been transferred to a solid substrate (Figure 1e). In this intermediate Π-regime, the size of clusters continuously grew until the interconnection between clusters emerged, which is typically regarded as a strong hallmark of the colloidal gel or crystal formation.^35,36^ Interestingly, unlike the report by Geisel et al.,^7^ where they found that charged microgels can be compressed more easily than uncharged ones, we clearly observed a superposition of the normalized isotherms (Π ≤ 30 mN/m) for microgels at different charged states (by varying pH or even temperature), except for one condition (pH 11, 24 °C). Further compression led to diverse behaviors for the second stage. This diversity exactly manifests the different pathways for the attractive colloidal crystallization^37–39^ during the second rapid increase of Π at varied conditions.

Next, inspired by the order-to-disorder transition by direct observation of the self-assembly at the interface, we performed a radial distribution function (RDF), *g*(*r*), to deduce the structural parameters, such as the nearest neighbor distance (Λ) (Figure S3). Representatives of *g*(*r*) at varied Π, pH, and temperatures are given in Figure 2 (see Figure S4 for more information). The first peak in *g*(*r*) corresponds to the probability of finding a particle at Λ or Λ/*a*, where *a* is the hydration diameter at each condition (Table S1). Typically, Λ/*a* decreased with the increase of Π and dropped suddenly around 20–25 mN/m, indicating the extensive formation of clusters at this threshold (see Figure 1e). At even higher Π, *g*(*r*) oscillated damply with *r*/*a*, where more than one peak could be identified (Figures S5–S10), indicating the strong signature of a hexagonal close-packed (hcp) lattice at least at short-range (Figure 1e, right).

**Figure 2:**
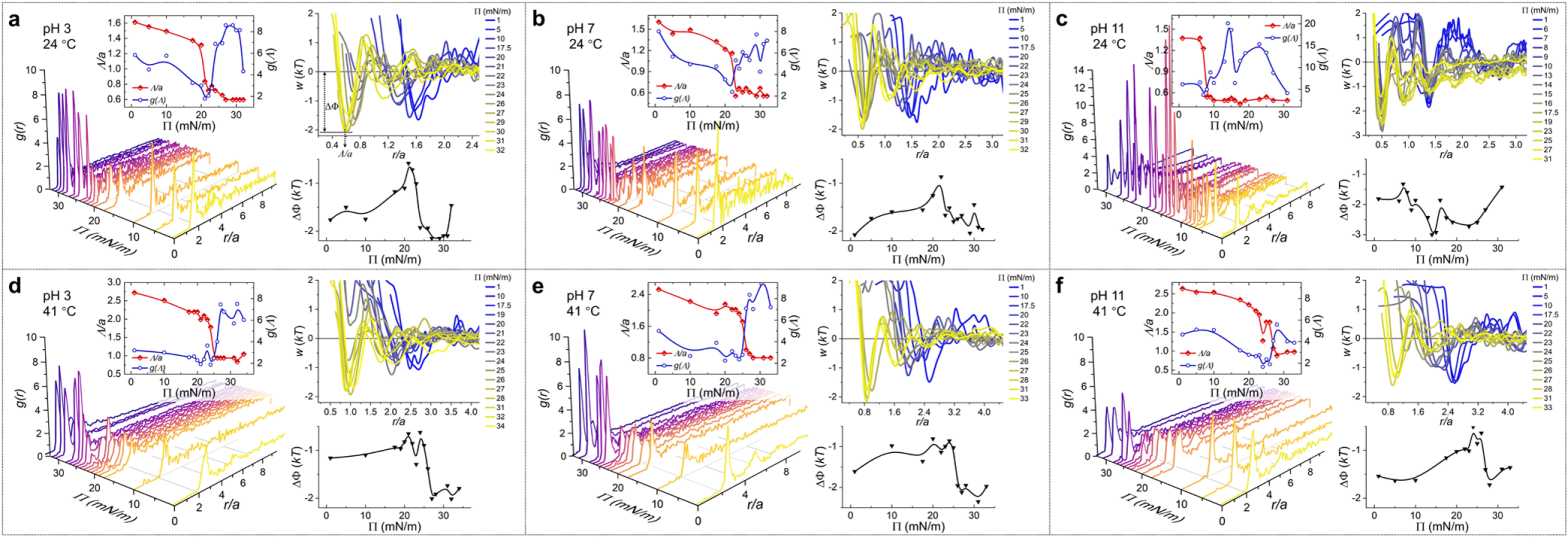
Representative plots of the radial distribution function *g*(*r*), nearest neighbor distance Λ, potential energy profiles *w*(*r*), and binding energy ΔΦ at various conditions: (a) pH 3, 24 °C, (b) pH 7, 24 °C, (c) pH 11, 24 °C, (d) pH 3, 41 °C, (e) pH 7, 41 °C, (f) pH 11, 41 °C. Dotted lines in the plot of *w*(*r*) vs *r*/*a* in (a) indicate the binding energy ΔΦ at corresponding Λ/*a*. See Figures S5–S10 for single RDF curves.

Meanwhile, we obtained the interparticle potential energy (*w*(*r*)) of mean force by Boltzmann inversion of *g*(*r*), i.e., *w*(*r*) = −*kT* ln­[*g*(*r*)], where *k* is the Boltzmann’s constant and *T* is the absolute temperature.^37,40–42^ Subject to uncorrected many-body correlations, the experimentally accessible *w*(*r*) employed here was referred to as an estimate for the real pair potential in the limit of infinite dilution. Under such criteria, the deepest minima in interaction energy (ΔΦ), corresponding to the binding energy of two microgels at Λ, identified the average core–core separations, which can locate at a distance smaller than *a*.

To better illustrate the variation trends of Λ/*a*, *g*(Λ), and ΔΦ, depending on Π at varied pH and temperatures, we summarized the above parameters. For the nearest neighbor distance Λ/*a* (Figure 3a), we clearly confirmed its decreasing trends with increasing Π, which has been described above. Meanwhile, Λ/*a* showed distinct dependences on the temperature, while the individual dependence was tuned slightly by pH. A particular case at pH 11 and 24 °C emerged again, where Λ/*a* showed a sudden reduction at a relatively low Π (i.e., ∼8 mN/m). The special behavior echoed with the compression isotherm and early cluster formation at an identical condition (see Figure 1d,e). Besides, Λ/*a* showed a mean value of ∼1.5 and 2.5 at low-Π regimes and ∼0.5 and 1 at high-Π regimes at 24 and 41 °C, respectively. Although the shape of the microgels would be stretched to a “fried-egg”-like structure^1^ by interfacial stress, the mean Λ/*a* can still be referred to as an effective parameter to unveil the interparticle arrangement at interface (Figures 3d and S11). In case I (i.e., Λ/*a* ∼ 1 at high Π and high *T*), the extended coronae at the air side interpenetrate deeply, while the collapsed and highly charged (ζ ∼ −27 mV) core area underwater expels to each other. Case III, i.e., Λ/*a* ∼ 0.5 at high Π and low *T*, shares similarities with case I but with a high compression of microgels, giving rise to the formation of an attractive colloidal crystal (see hcp lattices in Figure 1e, e.g., 24 °C, pH 7, 32 mN/m). For case II (i.e., Λ/*a* ∼ 2.5 at low Π and high *T*), a long-ranged ordering has been observed (see dotted arrows in Figure 1e, e.g., 41 °C, pH 7, 1 mN/m), indicating a non-close-packed repulsive crystal phase. Lastly, in case IV (i.e., Λ/*a* ∼ 1.5 at low Π and low *T*), considering the swelling of the thus fully stretched corona, we proposed that the coronae might be slightly entangled with a core–core (EDL) repulsion.

**Figure 3:**
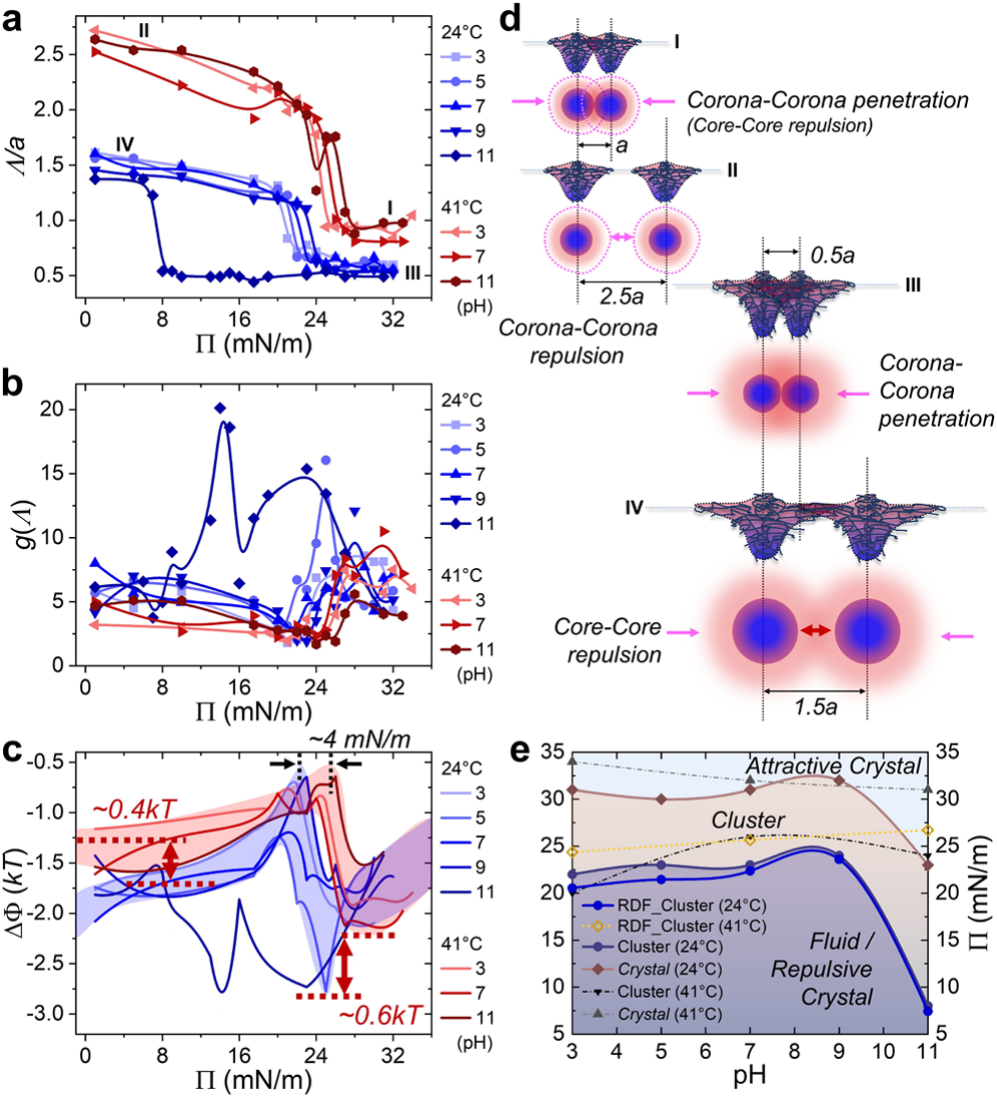
(a–c) Plots of the nearest neighbor distance Λ (a), radial distribution function *g*(Λ) (b), and binding energy ΔΦ (c) as functions of Π. Blue and red shadows in (c) denote the band-like structures at 24 and 41 °C, respectively. (d) Schematic representations of two swollen or collapsed microgels at the interface for cases I–IV. Corresponding conditions are denoted in (a). (e) Phase diagram plotted by direct observation of interfacial morphology or calculation of RDF (see Table 1).

Apart from the observations on a length scale, we further correlated the intermacromolecular interaction with the 2D phase behavior from an energic standpoint (Figure 3b,c). By classifying the binding energy ΔΦ vs Π according to temperature, we clearly observed two distinct band-like structures, in analogy to solid-state theory, where the “energy level” was refined by pH. The *kT*-scaled binding energy (or attractive strength) between neighboring microgels (corresponding to intercorona or intercore interaction) at a low temperature was measurably stronger than that at a high temperature (i.e., by ∼0.4*kT* at low Π and by ∼0.6*kT* at high Π). The steep decrease of ΔΦ around 20–25 mN/m, similar to that of Λ/*a*, further examined the early stage formation of small-sized clusters (corresponding to liquid-/repulsive crystal-to-cluster transition), which emerged at a relatively lower Π for systems at a low temperature.

Integrating the above phenomena and analysis led to a simple phase diagram for the ionizable and deformable microgel system (Figure 3e and Table 1). Briefly, the microgel system showed a coexistence of the ordered and disordered phases, which underwent a fluid or repulsive crystal to cluster and then to attractive colloidal crystal transition. The interphase boundaries can be finely tuned by pH, but will be totally redefined by temperature.

**Table 1:** Critical Surface Pressure Π Obtained from the Interfacial Morphology and RDF

	interfacial morphology				RDF	
	24 °C		41 °C		24 °C	41 °C
pH	Π_CLS_ ^a^ (mN/m)	Π_CC_ ^b^ (mN/m)	Π_CLS_ ^a^ (mN/m)	Π_CC_ ^b^ (mN/m)	Π_CLS_ ^a^ (mN/m)	Π_CLS_ ^a^ (mN/m)
3	22	31	20	34	20.55	24.37
5	23	30			21.45	
7	23	31	26	32	22.37	25.64
9	24	32			23.60	
11	8	23	24	31	7.43	26.71

a Critical Π for fluid-to-cluster transition.

b Critical Π for attractive colloidal crystal formation.

In the above discussions, the interparticle attraction governing interfacial self-assembly was primarily attributed to the intermacromolecular chain entanglements; however, the effect of capillary force could not be ignored and may play a key role, especially for deposition and dewetting processes.^43–45^ Near the three-phase (i.e., solid substrate–air–water film) contact line, clustering of microgels has been demonstrated by recent progress, which elaborately combined in situ and ex situ observations.^22,46–48^ Isostructural phase transition (IPT) has been examined to arise from the dewetting of deposited monolayers, particularly when the capillary force overcomes the adhesion force. Based on these findings, the phase diagram given in this study might not be accurate enough to fully describe the in situ self-assembly of microgels at liquid interfaces. Advanced technologies such as freeze-fracture shadow casting cryo-SEM^7,12,43,44^ and Langmuir–Schaefer deposition with supercritical drying^46^ are good alternatives, which have been developed recently to suppress the capillary effect, offering exciting opportunities to access the in situ interfacial structure and provide useful solutions for colloidal soft lithography.

On the other hand, regardless of characterizations ex situ or in situ, a critical transition has been confirmed very recently, where the nearest neighbor distance, Λ vs Π, and the hexagonal order parameter, Ψ_6_ vs Π, concurrently illustrate an identical turning point within the range of 20–25 mN/m.^46,47^ Our results (Figure 3a and Table 1, critical Π obtained from RDF) share similarities with the above findings. We speculate that such a critical transition originates from the intermacromolecular interaction switching between repulsive and attractive interactions under varied conditions, as illustrated in Figure 3d. The capillary force might amplify the intercorona attraction, leading to cluster formation after deposition. Nevertheless, the counterbalance between chain entanglement, steric hindrance, DLVO forces, and capillary effect for interfacial microgel self-assembly remains an open question.

In conclusion, we have explored interfacial behaviors of one model system containing dual pH–temperature responsive microgels from the multiscaled perspectives of length, structural parameter, and interaction energy. We focused on the complex interplay among the interfacial stress, two-dimensional self-assembly, phase behaviors, intermacromolecular interactions, and other environmental factors, such as pH and temperature. The experimental approach and the concept invoked here could be readily used for other macromolecular or colloidal systems, including but not limited to nano/microgels, coacervate, micelles, proteinoid microspheres, granular materials, etc.

## Supplementary Material



## Notes

Synthesis, sample preparation/characterization, interfacial measurements, and radial distribution function (PDF)
