# Supplementary material for: Intermacromolecular Interaction Determines the Long-Ranged Force and Self-Assembly of Microgels at the Air/Water Interface
Source: ACS Macro Lett. 2025 Apr 22;14(5):564–9. doi: 10.1021/acsmacrolett.5c00111 (PMC12096442; doi:10.1021/acsmacrolett.5c00111)
Supplement: Supplementary file 1 [file mz5c00111_si_001.pdf]

Supporting Information

Intermacromolecular Interaction Determines the Long-ranged Force and Self-assembly of Microgels at the Air/Water Interface

Wei Liu,*,† Zuwei Zhao,† Li Zhang,† Kangle Zhou,† Pui Wo Felix Yeung,‡ Hang Jiang,† Cheng Yang,† Yuwei Zhu,† and To Ngai*,‡

†The Key Laboratory of Synthetic and Biological Colloids, Ministry of Education & School of Chemical and Material Engineering, Jiangnan University, Wuxi 214122, China

‡Department of Chemistry, The Chinese University of Hong Kong, Shatin, N.T., Hong Kong 999077, China

Corresponding author
Prof. Wei Liu, email: weiliu@jiangnan.edu.cn
The Key Laboratory of Synthetic and Biological Colloids, Ministry of Education & School of Chemical and Material Engineering, Jiangnan University, Wuxi 214122, China

Prof. To Ngai, email: tongai@cuhk.edu.hk
Department of Chemistry, The Chinese University of Hong Kong, Shatin, N.T., Hong Kong 999077, China

Materials

N-isopropylacrylamide (NIPAM, >98%, Tokyo Chemical Industry, Japan), N,N'-Methylenebis(acrylamide) (BIS, 99%, Shanghai Aladdin Biochemical Technology, China), Methacrylic acid (MAA, CP, Sinopharm Chemical Reagent, China), Potassium persulfate (KPS, AR, Sinopharm Chemical Reagent, China) and Sodium dodecyl sulfate (SDS, ≥99%, Sinopharm Chemical Reagent, China) were used for synthesis without further purification. Ethanol (AR, Sinopharm Chemical Reagent, China) and Deionized water (18.2 MΩ·cm, Smart-S15, HHitech, China) were utilized to prepare spreading solutions for Langmuir tough. Sodium hydroxide (NaOH, AR, Sinopharm Chemical Reagent, China) and Hydrochloric acid (HCl, AR, content ~36%, Sinopharm Chemical Reagent, China) were used as received to prepare solutions with various pH.

Microgel Synthesis and Characterization

The microgel synthesis was done by a standard precipitation polymerization method with surfactant and was already described in detail elsewhere.[1-2] In brief, a mixture of NIPAM (4.356 g), BIS (0.304 g), MAA (0.315 g), and SDS (0.020 g) was dissolved in 148 mL D.I. water and put into a three-neck round-bottom flask equipped with a magnetic stirrer, a reflux condenser, and a nitrogen gas inlet. After stirring for 30 min at 70 ℃ under nitrogen purge, KPS (0.122 g) dissolved in 3 mL D.I. water was injected into the flask to initiate the polymerization. The reaction was maintained at 70 ℃ for 4 hours to achieve the p(NIPAM-*co*-MAA) microgels. To remove unreacted chemicals and surfactants, the dispersion was purified via filtration and dialysis for 4 days with daily twice replacement of D.I. water, and concentrated using a rotary evaporator (HR-21M, Shanghai Huxi Industry, China). Before measurements, the pH of the dispersions was adjusted by 0.1 M NaOH or 0.1 M HCl solution.

The hydrodynamic diameter D_h of the p(NIPAM-*co*-MAA) microgels ($c = 2 \times 10^{-4}$ g/mL) was determined by dynamic light scattering (ZetaPALS, Brookhaven Instruments Corporation, USA) at varied pH (3 to 11) and temperatures (20 to 44 °C). The pH and temperature dependences of the zeta potential ζ for the microgels were determined via a Zetasizer Nano (ZEN3700, Malvern Instruments Inc., U.K.) at $c = 1 \times 10^{-4}$ g/mL. The suspension was allowed to equilibrate at least for 5 minutes at each temperature before measurement, and all the test was conducted for three times at each condition. Microgel thin films were prepared by natural drying of the droplets of microgel dispersions ($c = 5 \times 10^{-3}$ g/mL, pH ranging from 3 to 11) at pre-cleaned microscopy slides. The air-water contact angles (WCA) of the microgel thin films were measured using a video optical contact angle meter (OCA15EC, DFE Chemie GmbH, Germany) (droplet volume: 3 μL).

Langmuir Trough

To observe the interfacial behavior, microgel monolayer at the air/water interface was prepared and compressed by a Teflon Langmuir trough (MicroTrough G2, Kibron Inc., Finland) equipped with Teflon barriers and a Wilhelmy plate. The compression isotherms were

measured with a compressible area of 27999 mm^2 (inner trough dimensions: width: 80 mm, length: 405 mm, depth: 5 mm). Before measurement, the solution was spread into the Langmuir trough, and the air–solution interfacial surface pressure was then calibrated when the solution temperature reaches the set ones. After calibration, the microgel suspensions were diluted to 1 × 10^{-3} g/mL at desired pH, and then mixed with ethanol as a spreading agent (volume ratio of solution : ethanol = 1 : 1). A certain volume of the mixture (~200 μL) was added to the air/water interface. The interface was equilibrated for 30 minutes before starting a compression test, thereafter the barriers were driven to compress the interface symmetrically at a constant speed of 5 mm/min.

Silicon wafers (Sinopharm Chemical Reagent, China) were cut into 10 mm × 10 mm pieces and underwent rinsing with water and ethanol, the slides were dried using nitrogen gas and subsequently cleaned for 5 minutes in an ultraviolet-ozone plasma cleaner (PDC-002, Harrick Plasma, USA) before use. To transfer the microgel monolayers formed at the air/water interface onto a solid substrate, the silicon wafer was dipped in water before spreading the microgels at the air/water interface and it was then pulled up through the microgel monolayer at the air/water interface by a small programmable dipper (LayerX (90), Kibron Inc., Finland). The barrier position was automatically adjusted to maintain the target surface pressure. The pull-up speed was 0.3 mm/min at an angle of ~40°. The array structures of the microgels were further observed using a scanning electron microscope (SEM, S-4800, Hitachi Limited, Japan) at an accelerating voltage of 3 kV.

Radial Distribution Function

A radial distribution function, $g_{ij}(r)$, describes the probability of a particle j with coordinates $\mathbf{r}_j$ locating within a thin shell of infinitesimal thickness δr at distance r from another particle i with coordinates $\mathbf{r}_i$. From the obtained SEM images, the coordinates of the microgels were firstly obtained by manual labelling of the images, then a two-dimensional radial distribution function (2D-RDF) can be computed according to

$$g^{2\mathrm{D}}(r) = \frac{1}{\rho^{2\mathrm{D}}}\left\langle \frac{1}{N}\sum_{i=1}^{N_i}\sum_{j\neq i}^{N_j}\delta(r - r_{ij})\right\rangle$$

where $r_{ij} = |\mathbf{r}_i - \mathbf{r}_j|$, N is the total number of particles present, $\rho^{2\mathrm{D}}$ is the two-dimensional number density of particles, and δ is the Dirac delta function. The distances were binned with inner and outer radii of $r + n\Delta r$ and $r + (n+1)\Delta r$ with $\Delta r = 2$–4 pixels during the numerical computation of the 2D-RDFs.

To reduce the artifacts by the finite size of images, a two-dimensional periodic boundary condition was utilized as an approximation in the computation of the 2D-RDFs.

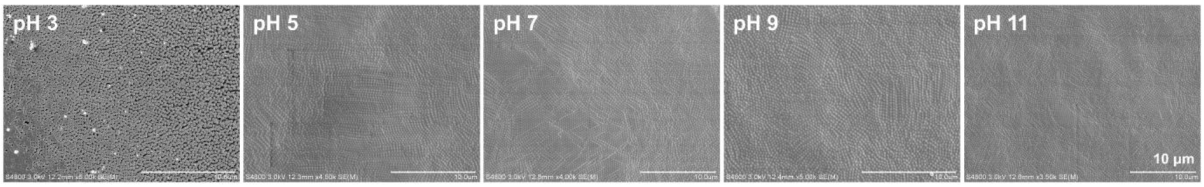

Figure S1. SEM images of the p(NIPAM-*co*-MAA) microgel films prepared at different pH for water contact angle measurements in Figure 1c. Scale bar: 10 μm.

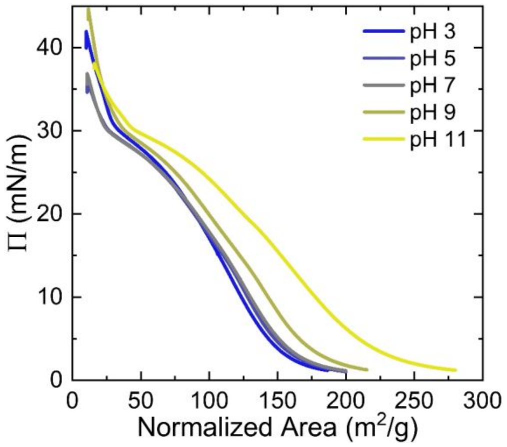

Figure S2. Compression isotherms of the p(NIPAM-*co*-MAA) microgels at the air/water interface at 24 °C and varied pH (3 to 11). The trough area was normalized by the amount of microgels at the interface.

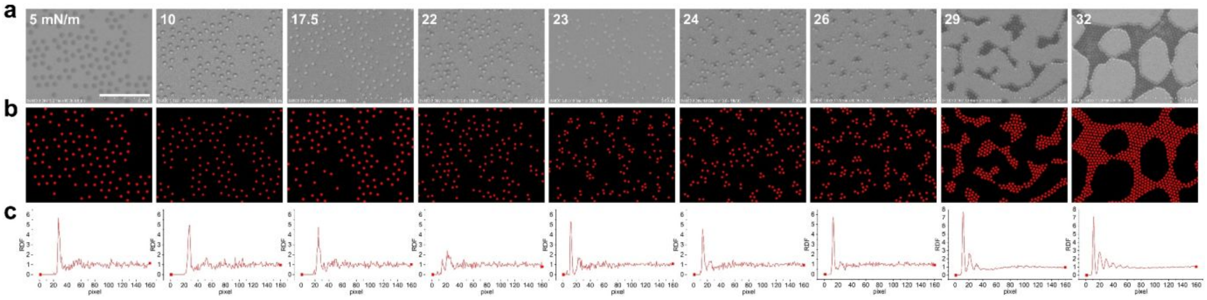

Figure S3. (a) SEM images of microgels at air/water interface after transfer to solid substrates at 24 °C and pH 7 (see Figure 1e). The magnitude of surface pressure in mN/m is indicated at the upper-left. Scale bar: 5 µm. (b) Regenerated array structures obtained by manual labelling. (c) Radial distribution functions (RDF) for the corresponding array structures in (b).

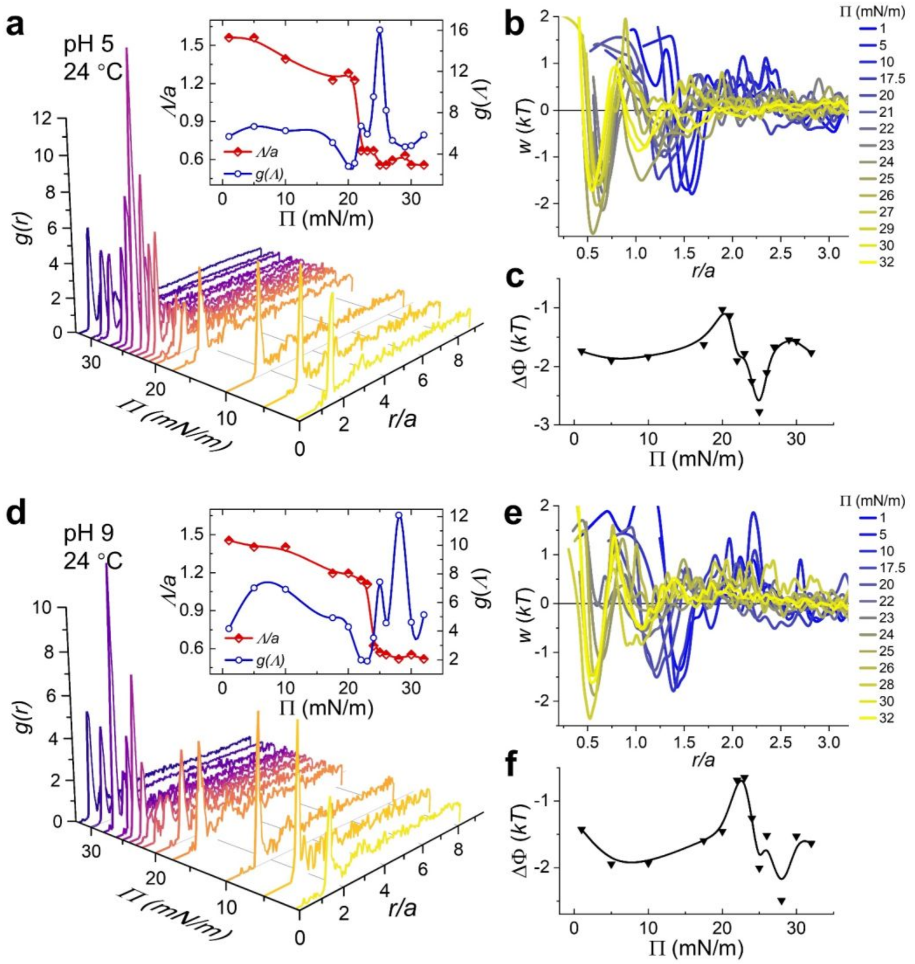

Figure S4. (a, d) Radial distribution function $g(r)$. Inset: nearest neighbor distance Λ/a. (b, e) Potential energy profiles $w(r)$. (c, f) Binding energy $\Delta\Phi$. Data were obtained at 24 °C and pH 5 (a–c) and pH 9 (d–f).

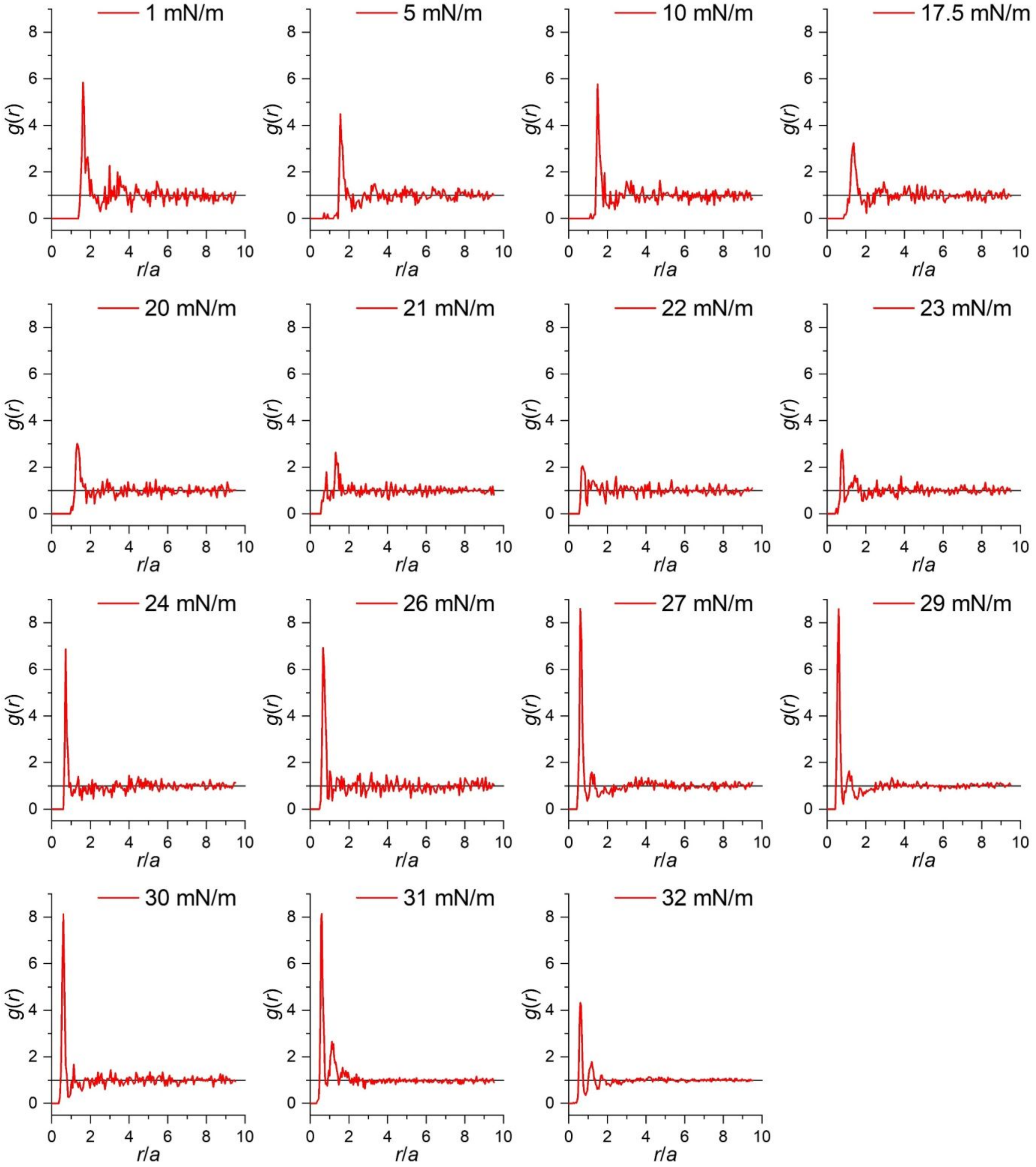

Figure S5. Single RDF curves for microgel packing at various surface pressure under conditions of pH 3 and 24 °C (corresponding to the RDF in Figure 2a).

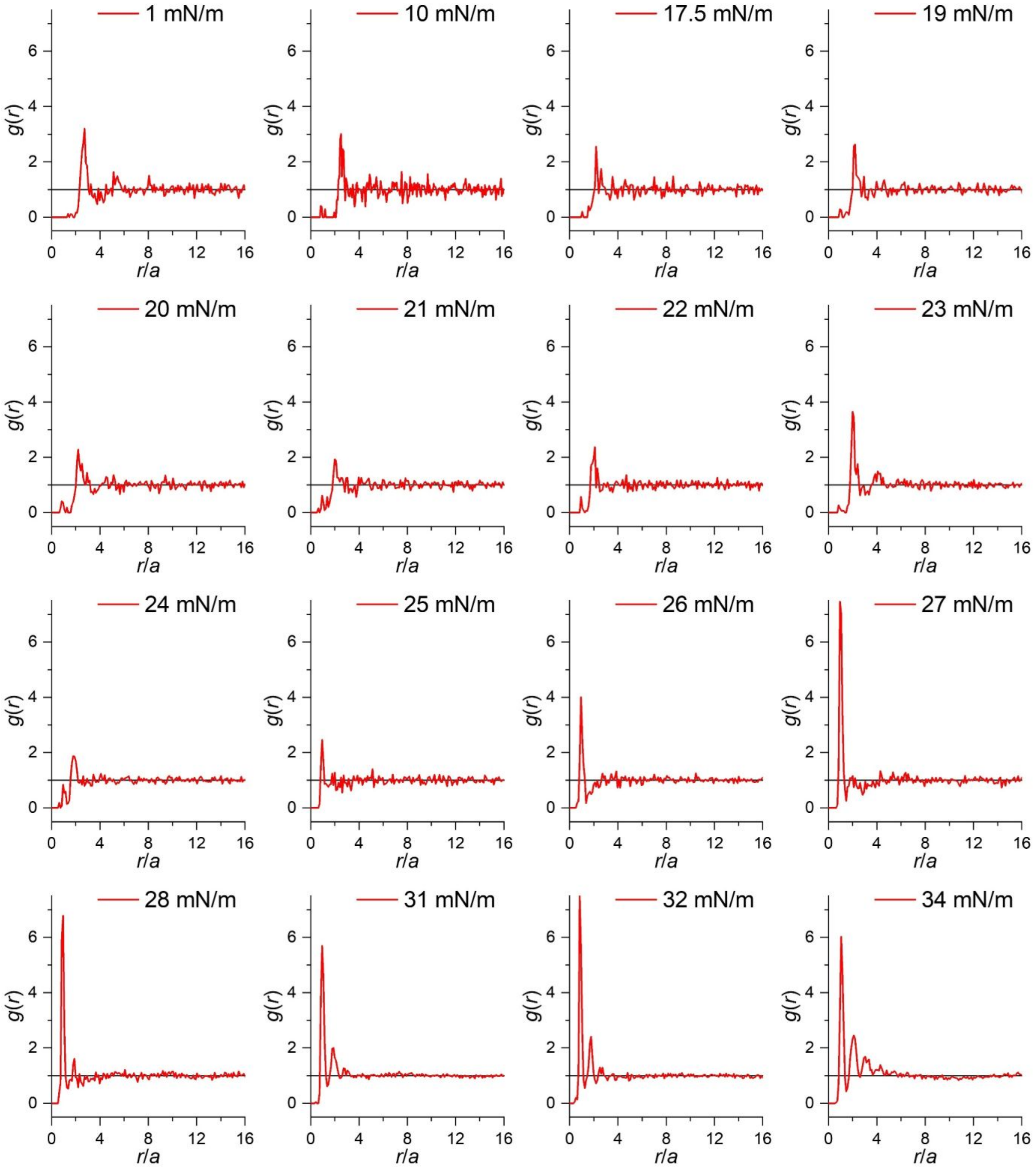

Figure S6. Single RDF curves for microgel packing at various surface pressure under conditions of pH 3 and 41 °C (corresponding to the RDF in Figure 2d).

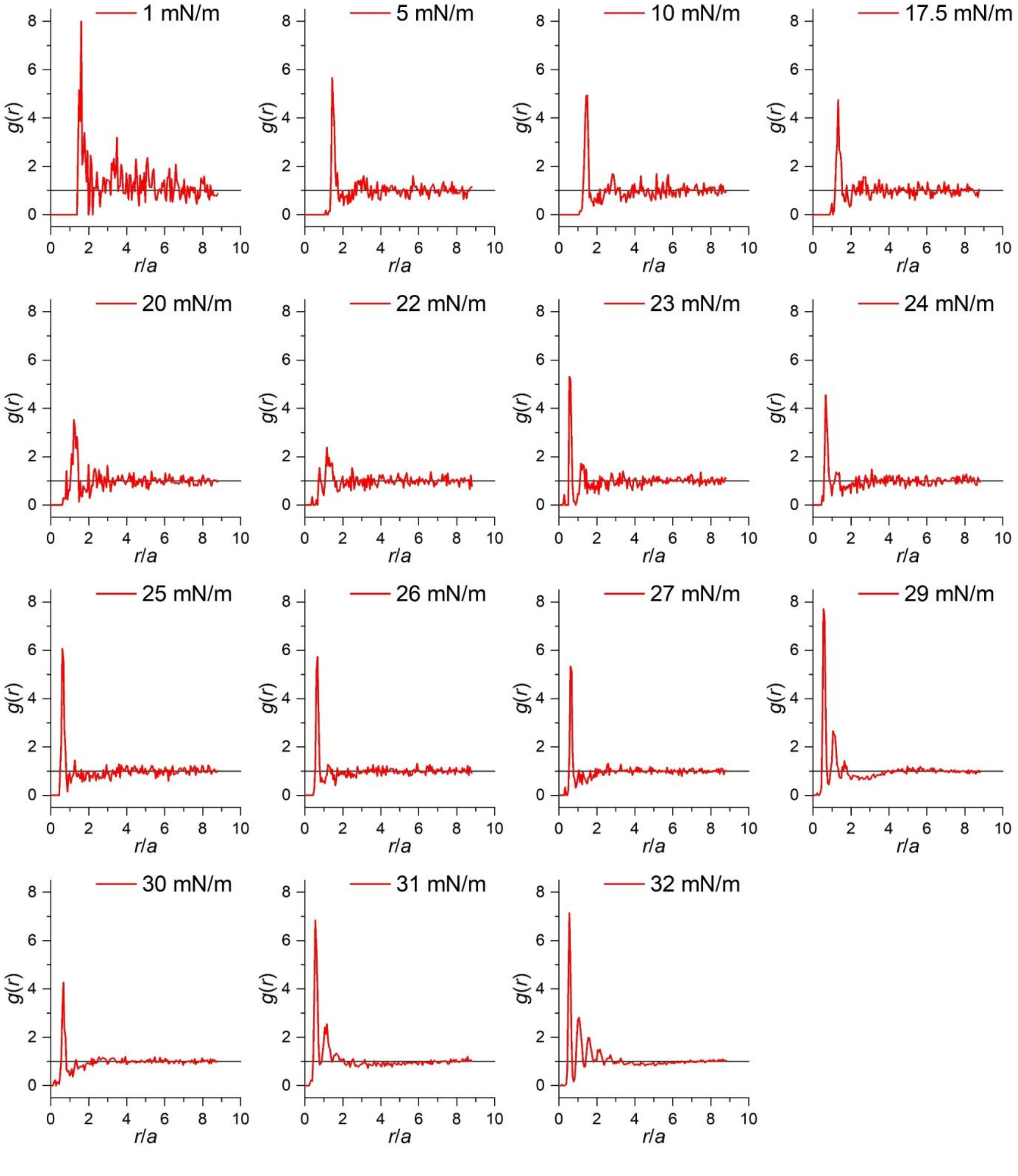

Figure S7. Single RDF curves for microgel packing at various surface pressure under conditions of pH 7 and 24 °C (corresponding to the RDF in Figure 2b).

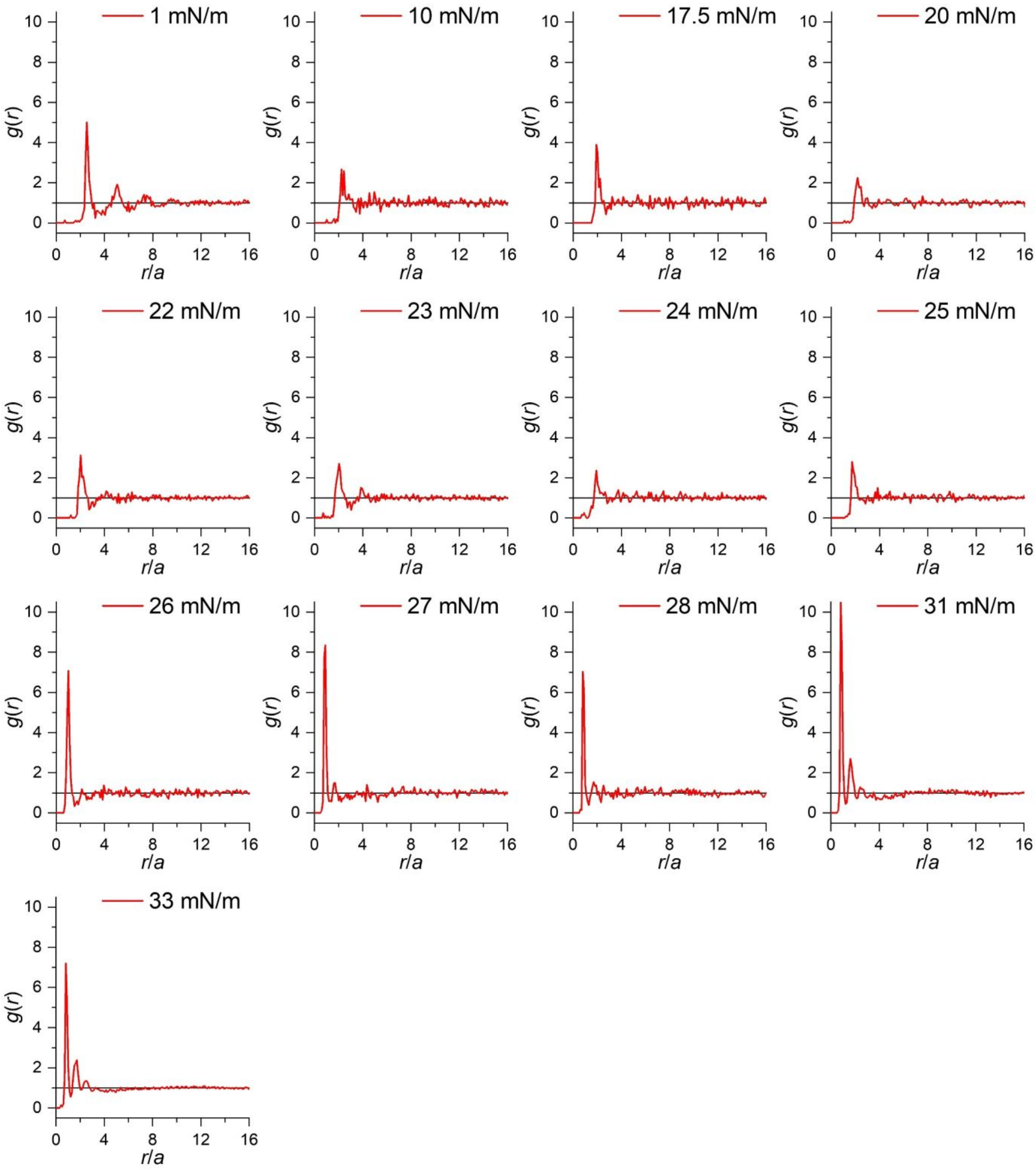

Figure S8. Single RDF curves for microgel packing at various surface pressure under conditions of pH 7 and 41 °C (corresponding to the RDF in Figure 2e).

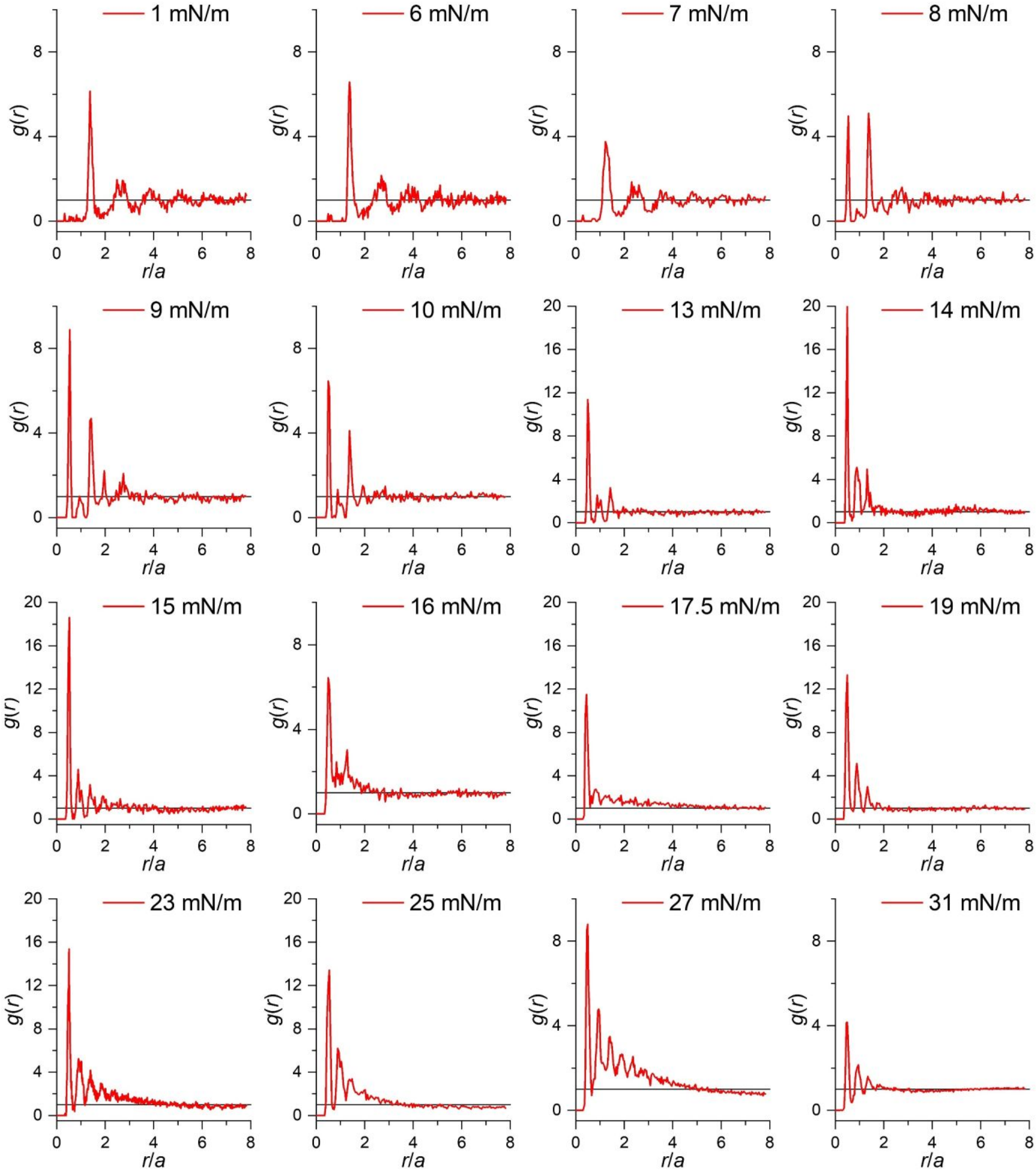

Figure S9. Single RDF curves for microgel packing at various surface pressure under conditions of pH 11 and 24 °C (corresponding to the RDF in Figure 2c).

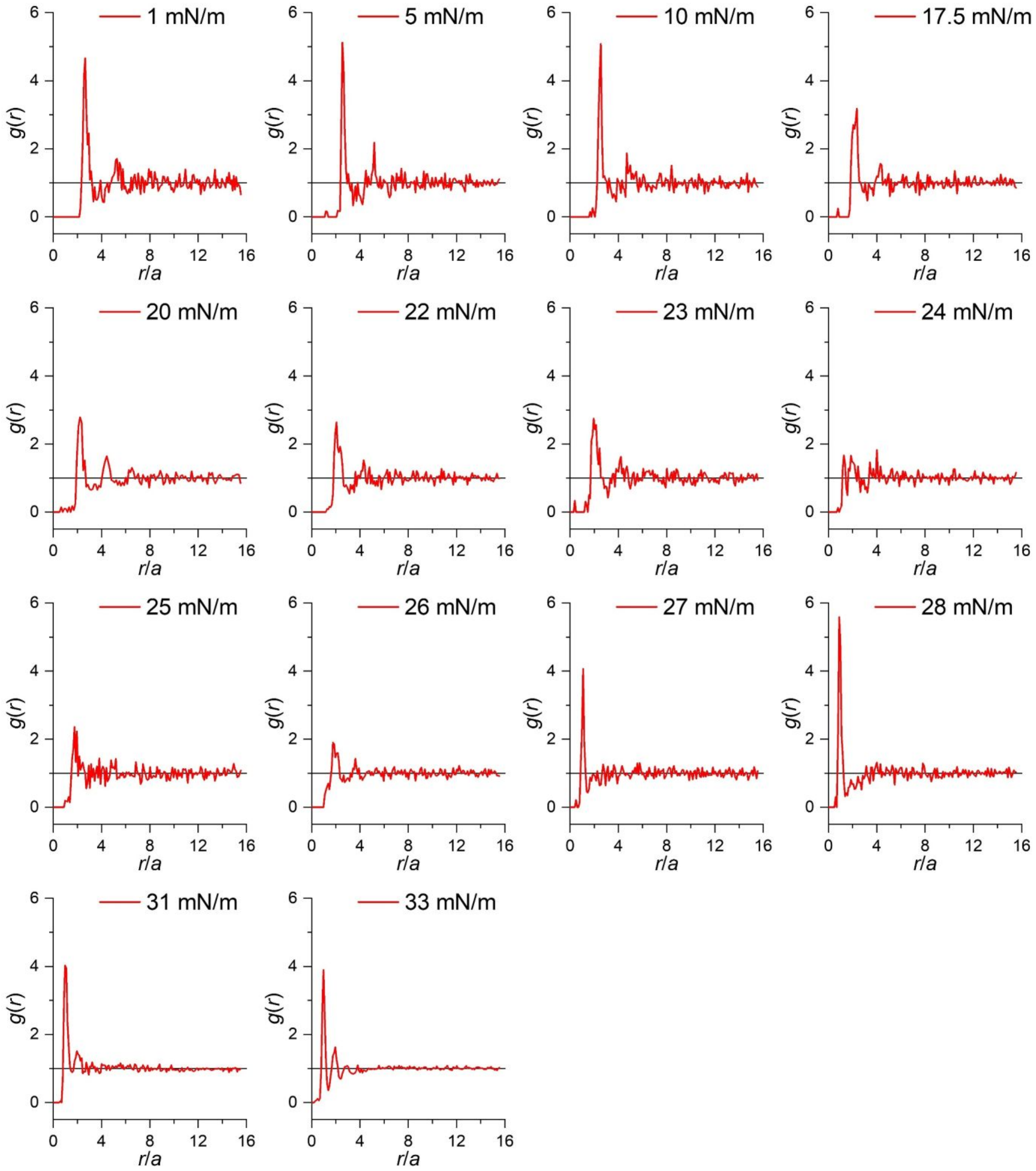

Figure S10. Single RDF curves for microgel packing at various surface pressure under conditions of pH 11 and 41 °C (corresponding to the RDF in Figure 2f).

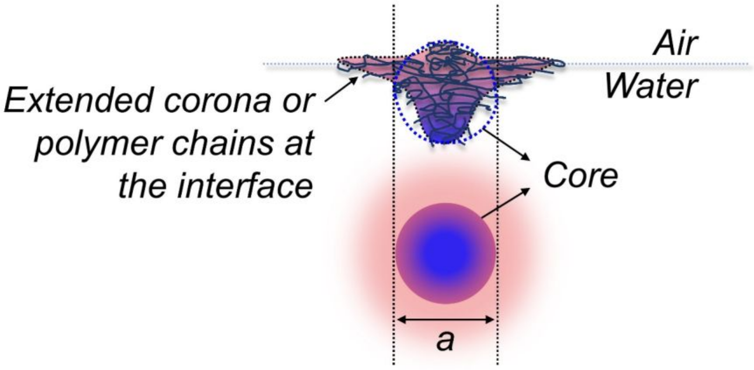

Figure S11. Schematic diagram of one single microgel at the air/water interface. Solid sphere and the blue dotted circle denote the “core” area with a hydration diameter of a or D_h (given by DLS). Red shadow denotes the extended “corona” area or confined polymer chains at the interface.

Table S1. Hydration diameter *a* of microgels at various pH and temperatures used in Figure 2, 3

	Hydration Diameter (nm)*	
pH	24°C	41°C
3	498.1	284.6
5	533.8	
7	538.0	294.7
9	572.7	
11	606.6	305.1

*Hydration diameters (D_h or a) were measured *via* DLS at each condition.

References

1. Zhou, S.; Chu, B., Synthesis and volume phase transition of poly (methacrylic acid-co-N-isopropylacrylamide) microgel particles in water. *The Journal of Physical Chemistry B* **1998,** *102* (8), 1364-1371.
2. Geisel, K.; Isa, L.; Richtering, W., The Compressibility of pH-Sensitive Microgels at the Oil–Water Interface: Higher Charge Leads to Less Repulsion. *Angewandte Chemie* **2014,** *126* (19), 5005-5009.
